# Rare earths stick to rare cyanobacteria: Future potential for bioremediation and recovery of rare earth elements

**DOI:** 10.3389/fbioe.2023.1130939

**Published:** 2023-02-28

**Authors:** Michael Paper, Max Koch, Patrick Jung, Michael Lakatos, Tom Nilges, Thomas B. Brück

**Affiliations:** ^1^ Werner Siemens-Chair of Synthetic Biotechnology, School of Natural Sciences, Department of Chemistry, Technical University of Munich, Garching, Germany; ^2^ Synthesis and Characterization of Innovative Materials, School of Natural Sciences, Department of Chemistry, Technical University of Munich, Garching, Germany; ^3^ Integrative Biotechnology, University of Applied Sciences Kaiserslautern, Pirmasens, Germany; ^4^ TUM AlgaeTec Center, Ludwig Bölkow Campus, Department of Aerospace and Geodesy, Taufkirchen, Germany

**Keywords:** cyanobacteria, biosorption, mechanism, rare earth elements, ion exchange

## Abstract

Biosorption of metal ions by phototrophic microorganisms is regarded as a sustainable and alternative method for bioremediation and metal recovery. In this study, 12 cyanobacterial strains, including 7 terrestrial and 5 aquatic cyanobacteria, covering a broad phylogenetic diversity were investigated for their potential application in the enrichment of rare earth elements through biosorption. A screening for the maximum adsorption capacity of cerium, neodymium, terbium, and lanthanum was conducted in which *Nostoc* sp. 20.02 showed the highest adsorption capacity with 84.2–91.5 mg g^-1^. Additionally, *Synechococcus elongatus* UTEX 2973, *Calothrix brevissima* SAG 34.79, *Desmonostoc muscorum* 90.03, and *Komarekiella* sp. 89.12 were promising candidate strains, with maximum adsorption capacities of 69.5–83.4 mg g^-1^, 68.6–83.5 mg g^-1^, 44.7–70.6 mg g^-1^, and 47.2–67.1 mg g^-1^ respectively. Experiments with cerium on adsorption properties of the five highest metal adsorbing strains displayed fast adsorption kinetics and a strong influence of the pH value on metal uptake, with an optimum at pH 5 to 6. Studies on binding specificity with mixed-metal solutions strongly indicated an ion-exchange mechanism in which Na^+^, K^+^, Mg^2+^, and Ca^2+^ ions are replaced by other metal cations during the biosorption process. Depending on the cyanobacterial strain, FT-IR analysis indicated the involvement different functional groups like hydroxyl and carboxyl groups during the adsorption process. Overall, the application of cyanobacteria as biosorbent in bioremediation and recovery of rare earth elements is a promising method for the development of an industrial process and has to be further optimized and adjusted regarding metal-containing wastewater and adsorption efficiency by cyanobacterial biomass.

## 1 Introduction

Rare Earth Elements (REE) consist of scandium, yttrium, and 15 elements of the lanthanide series. These elements have exceptional electromagnetic, catalytic, and optical properties making them crucial for the production and development of modern high-technology products. Due to their similar chemical properties, separating REE demands sophisticated industrial processes that are energy-intensive and use environmentally toxic chemicals (Haque et al., 2014). Standard methods, for example, apply metal leaching with acids or bases and extraction methods to purify REE (Opare et al., 2021). Moreover, REE production is focused on a few countries, resulting in an oligopoly that can dictate supply and price regimes. REE are crucial for technology transition towards a renewable energy-driven society. For instance, cerium or lanthanum have applications in catalysts for air purification or chemical processing. Other metals like neodymium or terbium are crucial for producing permanent magnets or modern LEDs (Charalampides et al., 2015; Shan et al., 2020). Hence, industrialized countries increasingly focus on alternative supply routes and the development of cost and ecologically compatible recycling routes. In this context, REE recovered from dilute mining or industrial wastewater, as well as, electronic waste streams are opening new, regional supply routes. Establishing new biotechnologically based REE recovery methods therefore leads to enhanced market stability and supply chain independence for industrialized regions, such as the EU. Hence, there is a growing interest in the recovery and recycling of REE from industrial wastewater streams (Li et al., 2013; Barros et al., 2019). Over the past decades, biosorption has been regarded as a relatively simple and cost-efficient method for wastewater treatment (Volesky 2001). It is a physicochemical process that involves a solid phase (biosorbent) consisting of organic biomass and a liquid phase containing the dissolved or suspended chemical compounds to be sorbed (sorbate) (Fomina and Gadd 2014). Biosorption has a wide range of potential applications in wastewater remediation, including the removal of organic substances like dyes, pharmaceuticals, or pesticides (Bell and Tsezos 1987; Aksu 2005; Crini and Badot 2008; Menk et al., 2019). However, most research on biosorption in conjunction with the removal of pollutants has been conducted on metals, including heavy metals, actinides, and lanthanides (Dhankhar and Hooda 2011; Abbas et al., 2014; Giese 2020; Mattocks and Cotruvo 2020). Yet, developed processes based on biosorption have not achieved a commercial breakthrough. For example, it has been shown that environmental factors, such as changes in the pH value, can alter the affinity of biomass towards different elements (Zinicovscaia et al., 2019). A low technology readiness level, including a poor understanding of the underlying mechanisms, kinetics, and thermodynamics of the process are areas that require more research (Fomina and Gadd 2014; Elgarahy et al., 2021). It is widely accepted that the chemical structure, in particular the composition of functional groups on the cell surface, profoundly influences the adsorption properties of biomass (Eccles 1999; Volesky 2007). These active moieties may include hydroxyl-, carboxyl-, carbonyl-, phosphate-, sulfonate-, amine-, amide-, and imide-groups, among many others. Studies on biological, physical, or chemical modification of biomass by adding functional groups have shown that it is possible to improve binding specificity and capacity for target sorbates (Wang and Chen 2006; Park et al., 2010; Abdolali et al., 2015; Ciopec et al., 2020). Especially the recovery of REE with chemically modified organic polymers has been the focus of recent studies (Gabor et al., 2017; Negrea et al., 2018; Negrea et al., 2020). Nevertheless, these resulting biosorbents are still inferior in target selectivity to chemically synthesized ion-exchange resins with a defined structure and composition (Gadd 2009). Due to the heterogeneity of functional groups on the cell surface of microbial biomass, binding specificity for elements remains a challenging factor for industrial applicability. The adsorption of heavy metals by eukaryotic algae and cyanobacteria is well documented (Al-Amin et al., 2021; Ankit et al., 2022). At present, the screening of new species regarding biosorption and potential novel applications in metal recovery remains of great interest due to high variability in cell wall composition and structure, resulting in differences in adsorption properties [e.g. (Micheletti et al., 2008)]. Cyanobacteria have shown promising adsorption properties for heavy metals, which could be used in the sequestration of metals from water on a technical scale. If similar adsorption properties exist for the bioremediation of REE has not been studied extensively yet. Moreover, the adsorption properties of terrestrial cyanobacteria were seldom investigated. Therefore, we taxonomically and biotechnologically identified new and promising cyanobacterial strains and evaluated their properties for REE adsorption. In this context, we also aimed to correlate taxonomic identity and adsorption characteristics. In this study, 12 cyanobacterial strains with broad phylogenetic origin and inhabiting different ecological habitats such as terrestrial, freshwater, and saltwater habitats were investigated for their potential applicability in an adsorption process for the enrichment of REE. Their phylogenetic relationship was determined using 16S rRNA sequences. The screening for maximum adsorption capacity with four different REE (i.e. lanthanum, cerium neodymium, and terbium), as well as the effect of several parameters on biosorption, including initial pH value, incubation time, and metal concentration for cerium, were evaluated. Additionally, binding specificity for cerium in the presence of other metal cations was investigated.

## 2 Materials and methods

### 2.1 Cultivation of cyanobacteria

12 cyanobacterial strains with broad phylogenetic origins and from different habitats were used for the study (Table 1). The cultures were inoculated with approximately 0.1–0.3 g of wet biomass from a stock culture (stored at 17°C and light, dark rhythm 16:8 h at 30 μmol photons m^−2^ s^−1^) in 1 L bubble columns containing BG11 cultivation medium (Stanier et al., 1971) (or Spirulina-medium for *Limnospira*) (Andersen 2005) and cultivated at 23°C and light, dark rhythm 16:8 h at 300 μmol photons m^−2^ s^−1^ photosynthetic photon flux density). All cultivated cells were harvested using filters (two sieves of 0.5 mm and 0.1 mm and finally paper filters of 40 µm openings), and wet biomass was dried by lyophilization. *Synechococcus elongatus* UTEX 2973 was cultivated in a 3.7 L Labfors 5 Photobioreactor (Infors GmbH, Sulzemoos, DE) in BG11 medium at 37°C and constant illumination at 300 μmol photons m^−2^ s^−1^. During cultivation, the pH was kept at eight by adding CO_2_ gas. Biomass was harvested by centrifugation after reaching the stationary phase.

**TABLE 1:** Overview of all investigated cyanobacteria strains in this study.

Strain	Strain number	Origin	Isolator, year	Order
*Nostoc.* (sp.)	20.02	Epiphytic on lichen *Peltigera* sp.; Germany	B. Büdel, 2000	Nostocales
*Synechococcus elongatus*	UTEX 2973	Freshwater, United States	J.Yu, 2011	Synechococcales
*Desmonostoc muscorum*	90.03, PCC 7906	Soil, United States	F. E. Allison, before 1930	Nostocales
*Calothrix brevissima*	SAG 34.79	Freshwater, Asia	Watanabe, before 1969	Nostocales
*Komarekiella* sp	89.12	Hypolithic on quartz, Namibia, South Africa	B. Büdel 1989	Nostocales
*Limnospira maxima*	SAG 49.88	Marine, Italy, Europe	Unknown, before 1988	Oscillatoriales
*Limnospira platensis*	SAG 85.79	Natron lake, Chad, Africa	G. Laporte, 1963	Oscillatoriales
*Phormidium autumnale*	97.20	small brook, polluted with sewage, Versasca Valley, Swiss, Europe	A. Zehnder, 1964	Oscillatoriales
*Komarekiella* sp	90.01	Chasmolithic in stone, South Africa	B. Büdel, 1990	Nostocales
*Reptodigitus* sp	92.01	Endophilic, South Africa	B. Büdel, 1992	Nostocales
*Symphyonema bifilamentata*	97.28	Soil, Switzerland	A. Zehnder, 1965	Nostocales
*Scytonema hyalinum*	02.01	Ephedaphic on arid soil, Namibia, Africa	S. Dojani, 2002	Nostocales

### 2.2 Phylogenetic characterization of strains

About 50 mg of biomass from all cultures were collected during stationary growth phase and used for gDNA extraction with the DNeasy PowerSoil Pro Kit (Quiagen, Hildesheim, Germany) following the manufacturer’s instructions. The 16S–23S ITS gene region was amplified by PCR in a 50 µL reaction using the primers Wil1 and Wil18 (Wilmotte et al., 1993) and ready-to-go PCR mini beads (GE Healthcare, Chicago, United Sates) in a MiniAmp Plus Thermal Cycler (Thermo Fisher Scientific, Waltham, United Sates). PCR products were checked by gel electrophoresis using 1% (w, v) agarose and the E-Gel Power Snap Electrophoresis System (Invitrogen, Waltham, United Sates). Subsequently, PCR products of the expected length were purified with NucleSpin Gel and PCR Clean-up Kit (Macherey-Nagel GmbH & Co. KG, Düren, Germany) following the DNA and PCR cleanup protocol and sent for Sanger sequencing to Genewiz, Azenta (Germany GmbH, Leipzig, Germany) using the primers Wil1, Wil4, Wil5, Wil10, Wil11, Wil16, and Wil18 (Wilmotte et al., 1993). The generated sequences were assembled with Geneious Prime (v2021.0.1) software package (Biomatters Limited, New Zealand) and compared to already submitted sequences of those strains from public culture collections in terms of authenticity using the BLAST tool of the National Center for Biotechnology Information (NCBI) GenBank. Sequences of strains that are novel were submitted to GenBank, and their accession numbers are given in the phylogenetic tree. The assembled 16S rRNA gene sequences and related sequences of cyanobacterial strains cited from GenBank were used for phylogenetic analyses, including *Gloeobacter violacaeus* as outgroup for the 16S rRNA gene alignment, applying the Muscle algorithm in Mega X (Kumar et al., 2018). The evolutionary model that was best suited for the database used was selected based on the lowest Akaike information criterion value and calculated in Mega X which was the RGT G + I model of nucleotide substitutions. The maximum likelihood method (ML) with 1,000 bootstrap replications was calculated with Mega X and Bayesian inference (BI) phylogenetic analyses, with two runs of eight Markov chains executed for one million generations with default parameters with MrBayes 3.2.1 (Ronquist and Huelsenbeck 2003). Each analysis reached stationarity (average standard deviation of split frequencies between runs <0.01) before the end of the run.

### 2.3 Metal analysis

The metal concentration in the analyzed solutions was determined via ICP-OES (Inductively Coupled Plasma Optical Emission Spectrometry) (Agilent 725 Series ICP Optical Emission Spectrometer, Agilent Technologies Inc., United Sates). A TraceCERT^®^ Rare earth element mix for ICP with 16 elements from Sigma-Aldrich (Sigma-Aldrich, Taufkirchen, Germany) and a Certipur^®^ ICP multi-element standard solution IV from Merck (Merck KGaA, Darmstadt, Germany) with 23 elements, were used as standards for calibration. Data analysis was done with ICP Expert II Agilent 725-ES Instrument Software Version 2.0 (Agilent Technologies Inc., United Sates).

### 2.4 Biosorption

Before the experiments, all biomass samples were washed three times with demineralized water to remove residual media components that could falsify the measurement results. The washed biomass was frozen at −80°C and lyophilized. Sorption experiments were carried out by incubating lyophilized biomass in metal solutions with a defined concentration. Each experiment was performed in triplicates. Metal uptake was determined by comparing the metal concentrations before and after incubation. Prior to measuring the metal concentration, each sample was centrifuged at 10,000 rcf for 5 min at room temperature. The supernatant was subsequently used for analysis. The adsorption experiments in this study predominantly focused on cerium, as it is the most prevalent REE.

### 2.5 Adsorption capacity

Adsorption experiments were performed based on a methodology described in previous studies (Heilmann et al., 2015; Heilmann et al., 2021). To determine the metal adsorption capacity (Q) of the different strains, 10–20 mg of dry biomass of individual species were weighed into centrifuge tubes and incubated in 2 mL metal solutions for 3 h under constant shaking at room temperature. Subsequently, the adsorption of the metals to the biomass was determined by dividing the changes in metal concentration by the amount of incubated biomass (see Eq. 1).

$$Q=\frac{n_{i}-n_{f}}{m}=\frac{\left(c_{i}-c_{f}\right)\times V}{m}$$ (1)

with Q = adsorption capacity, n_i_ = initial amount of substance, c_f_ = final amount of substance after incubation, c_i_ = initial metal concentration, c_f_ = final metal concentration after incubation, V = volume, and m = weight of biomass.

For the determination of the maximal adsorption capacity during the screening, metal solutions with a concentration of 10 mM and an initial pH value of 5 ± 0.2 were used.

### 2.6 Adsorption kinetics

Experiments on adsorption kinetic were carried out by varying the incubation time of the biomass in cerium (III) nitrate solutions with a concentration of 10 mM and a pH-value of 5 ± 0.2. Samples for analysis were taken after an incubation time of 2 min, 5 min, 15 min, 30 min, and 60 min.

### 2.7 Effect of initial pH value

The influence of the pH value in metal biosorption was investigated similarly to the method previously described. The pH value of the applied metal solution was adjusted using hydrochloric acid and sodium hydroxide. Due to the formation of insoluble REE hydroxides at pH values above 7 (Plancque et al., 2003; Heilmann et al., 2021) the experiments were carried out ranging from pH 1 to 6.

### 2.8 Adsorption isotherms

Adsorption isotherms were studied by varying the metal concentration of solutions applied to the biomass samples between 0.5 and 10 mM. Samples were incubated for 1 h at room temperature under constant shaking and analyzed as previously stated. The adsorption isotherms were described using the Langmuir and Freundlich model (see Eqs. 2, 3). The Langmuir model is often used for the description of metal adsorption as it assumes adsorption in the form of a monolayer onto a surface containing a finite number of identical binding sites (Dada et al., 2012). By contrast, the Freundlich model assumes metal adsorption on non-identical bindings sites over a heterogeneous surface (Koong et al., 2013).

$${Langmuir\;model:\;Q}_{eq}=Q_{\max}\frac{K\times C_{eq}}{1+K\times C_{eq}}$$ (2)

$${Freundlich\;model:\;Q}_{eq}=K_{f}{C_{eq}}^{bf}$$ (3)

with Q_eq_ = adsorption capacity at equilibrium, Q_max_ = maximum adsorption capacity, K = Langmuir adsorption coefficient, K_f_ = Freundlich adsorption capacity constant, C_eq_ = metal concentration at equilibrium, and b_f_ = Freundlich isotherm constant.

Calculations for data analysis and model fitting were done using OriginPro 2020.

### 2.9 Adsorption specificity

Wastewater usually contains a mixture of different metal cations. In addition to examining the capacity for a single element of interest, it is therefore important to investigate whether some metal cations are adsorbed preferentially over others by the cyanobacterial biomass. Experiments were carried out with equimolar mixed-metal solutions with concentrations of 0.5–4 mM to investigate the binding specificity of the biomass for different metals. Following previous experiments on green algae and cyanobacteria by Klimmek (Klimmek 2003), the adsorption of cerium in the presence of aluminum, lead, nickel, and zinc was investigated. The uptake of metals by the biomass was measured using ICP-OES measurements, analogous to determining the adsorption capacity with single metal solutions.

### 2.10 FT-IR analysis

IR spectroscopy is a useful tool for the qualitative measurement of organic functional groups. In this study, IR spectroscopy was used to identify functional groups in cyanobacteria biomass samples and to detect possible interactions with metal cations. Samples were incubated in a cerium (III) nitrate solution (1 µmol 1 mg^-1^ biomass) for 2 h and subsequently lyophilized. IR spectra were recorded using a Nicolet iS50R FT-IR spectrometer from Thermo Fisher Scientific (Thermo Fisher Scientific, Waltham, United Sates) equipped with an iS50 ATR (Attenuated total reflection) multi-range, diamond sampling station. For each sample, IR spectra were obtained in a range from 400–4,000 cm^-1^.

## 3 Results

### 3.1 Phylogenetic analysis

**FIGURE 1:**
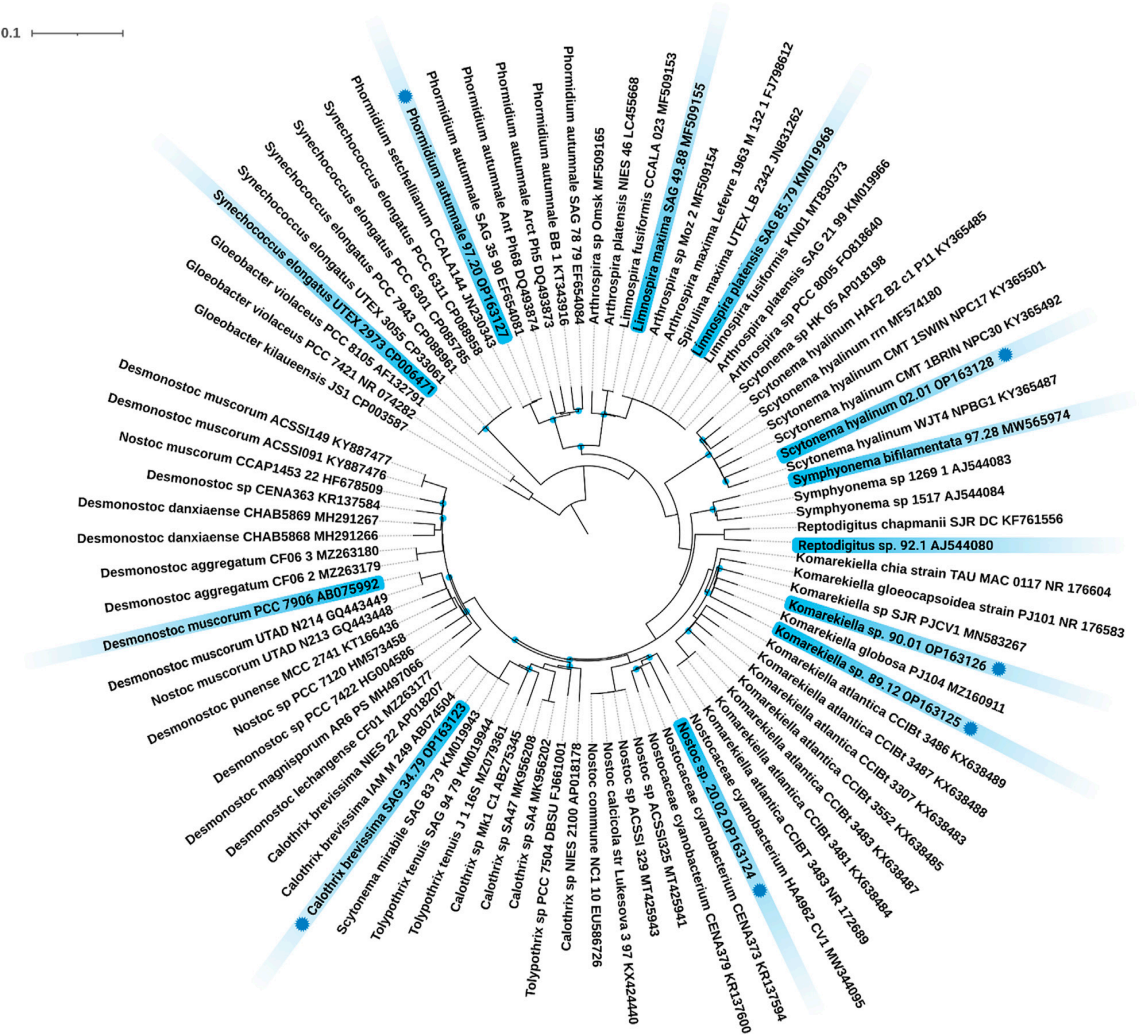
Maximum Likelihood (ML) phylogenetic tree based on the 16S rRNA gene region. Marked in blue are the twelve investigated strains including their strain number and NCBI accession number. Strains with a blue dot indicate novel 16S rRNA sequences generated during this study. Since the resulting Bayesian Inference (BI) and ML phylogenetic trees mostly showed the same topology, a single tree with both BI and ML bootstrap values is shown. Supports at the nodes greater than 90% statistical support from BI and ML represent posterior probabilities, and bootstrap values indicated as blue circles. The scale bar specifies 0.1 expected changes per site.

Twelve different cyanobacteria, including four strains from public culture collections and eight environmental isolates were investigated. The identity of all strains from public culture collections was confirmed based on their 16S rRNA sequence using the BLAST tool of GenBank. The 16S rRNA sequences of the strains 97.20, 02.01, 90.01, 89.12, 20.02, and SAG 34.79 were originally recovered, and their phylogenetic position was analyzed (Figure 1). In detail, strains 90.01 and 89.12 clustered well supported within the filamentous, heterocyte-forming genus *Komarekiella* while strain 20.02 clustered within the genus *Nostoc sens*. *lat*. The strain *Calothrix brevissima* SAG 34.79 joined a cluster of other *C. brevissima* strains and strains assigned to the genera *Tolypothrix* and *Scytonema* with 100% identity. Strain 02.01 fell well supported in the large cluster of *Scytonema hyalinum,* whereas strain 97.20 could be assigned to *Phormidium autumnale* based on its high similarity with 16S rRNA sequences from other filamentous, non-heterocyte forming strains representing this species. Thus, the cyanobacterial strains represent a broad phylogenetic origin out of the three orders Synechococcales, Oscillatoriales and Nostocales, inhabiting different ecological habitats such as terrestrial, freshwater, and saltwater habitats and most are new for biotechnological applications, particularly for their adsorption process of REE.

### 3.2 Screening for maximum adsorption capacity

**FIGURE 2:**
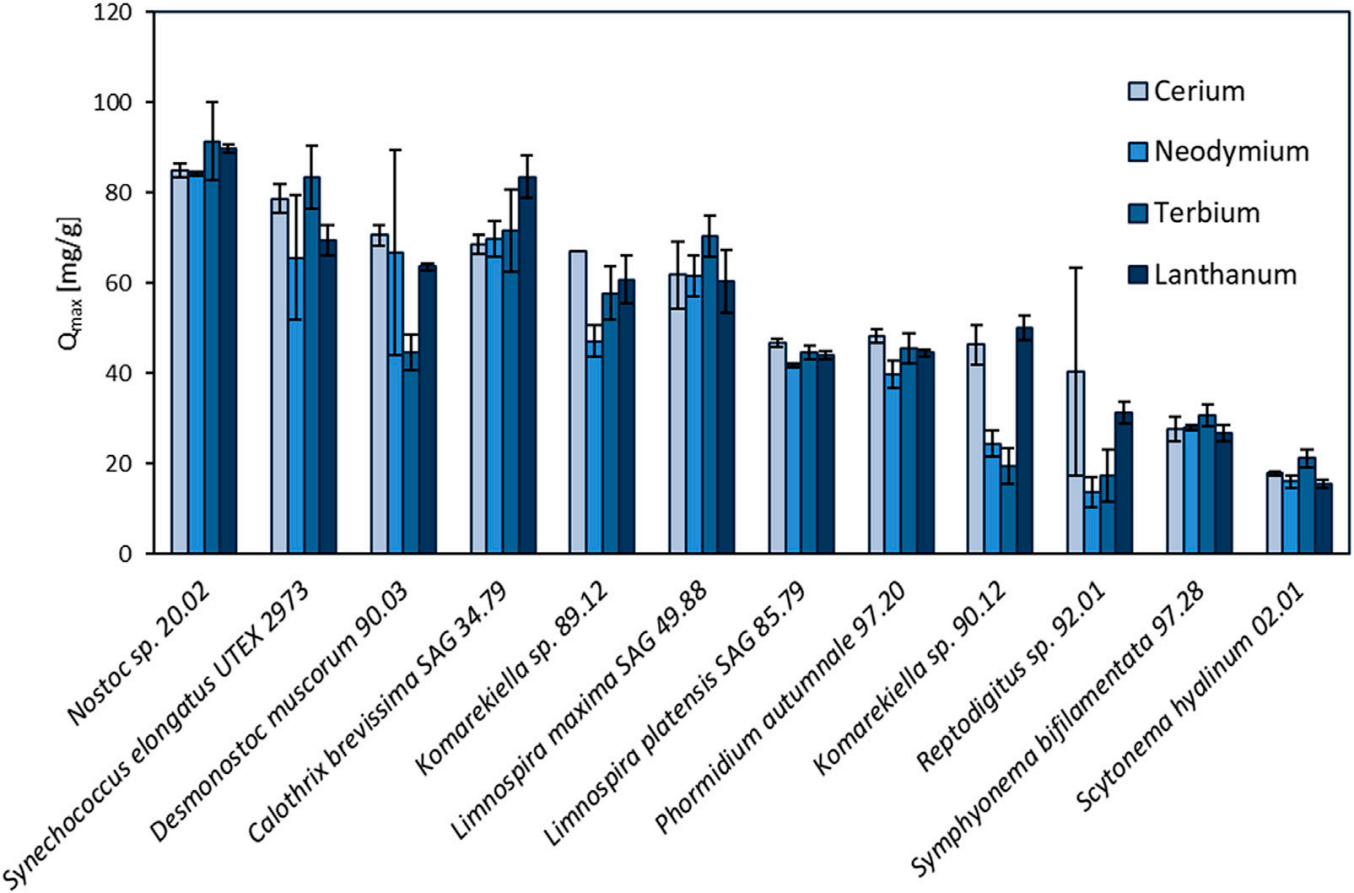
Screening of 12 different cyanobacteria for their maximum adsorption capacity (Q_max_, mg REE g^-1^ dry mass) of cerium, neodymium, terbium, and lanthanum (pH: 5 ± 0.2, n = 3).

In this study, the maximum adsorption capacity for REE (cerium, neodymium, terbium, and lanthanum) of 12 different cyanobacteria was investigated. The results of this screening are shown in Figure 2, depicting distinct differences in total metal uptake depending on the species. There was no apparent correlation between the capacity for REE adsorption for the phylogenetic relationship and the ecological habitat. The highest overall metal uptake of the four tested REE was observed for *Nostoc* sp. 20.02 adsorption capacities between 84.2 and 91.5 mg g^-1^, while *S. hyalinum* 02.01 exhibited the lowest maximum adsorption capacity with 15.5–21.2 mg g^-1^. Based on these results, the biosorption properties of the five most efficient cyanobacteria (*Nostoc* sp. 20.02*, Synechococcus elongates* UTEX 2973*, Desmonostoc muscorum* 90.03*, C. brevissima* SAG 34.79*,* and *Komarekiella* sp. 89.12) were investigated in more detail.

### 3.3 Effect of different initial pH values on metal adsorption

**FIGURE 3:**
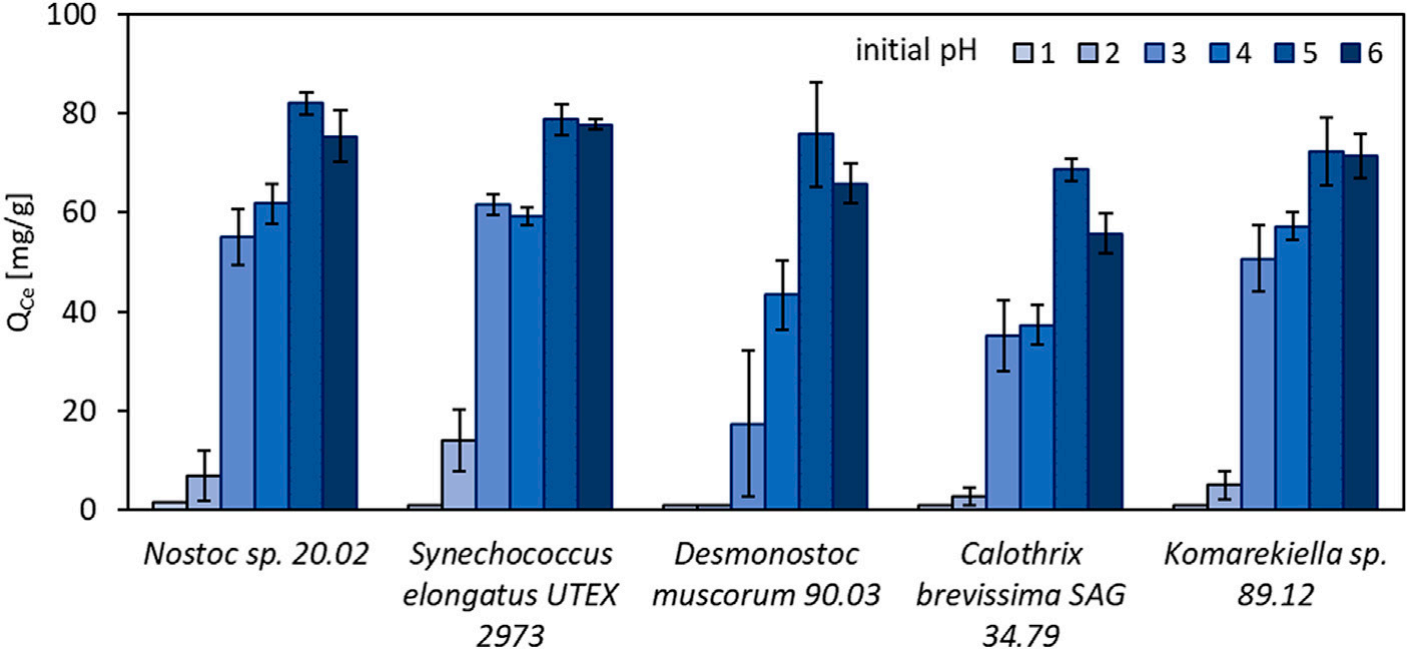
Adsorption capacity for Ce^3+^ (Q_Ce_, mg Ce^3+^ g^-1^ dry mass) of different cyanobacteria in solutions with initial pH values between 1 and 6 (n = 3).

The effect of the initial pH value of metal-solutions on biosorption of REE was examined in a pH range between 1 and 6. Experiments with 10 mM cerium (III) nitrate showed a strong influence of the pH value on metal adsorption (Figure 3). The highest metal uptake was observed at pH 5, with a minor decrease at pH 6. With increasing acidity, metal adsorption rapidly decreased. At pH 1, no notable metal adsorption was measured for all tested biomasses. These results are in accordance with previous studies on cyanobacteria, bacteria, and green algae regarding metal adsorption (Kuyucak and Volesky 1988; Gong et al., 2005; Lupea et al., 2012; Liang and Shen 2022).

### 3.4 Adsorption kinetics

**FIGURE 4:**
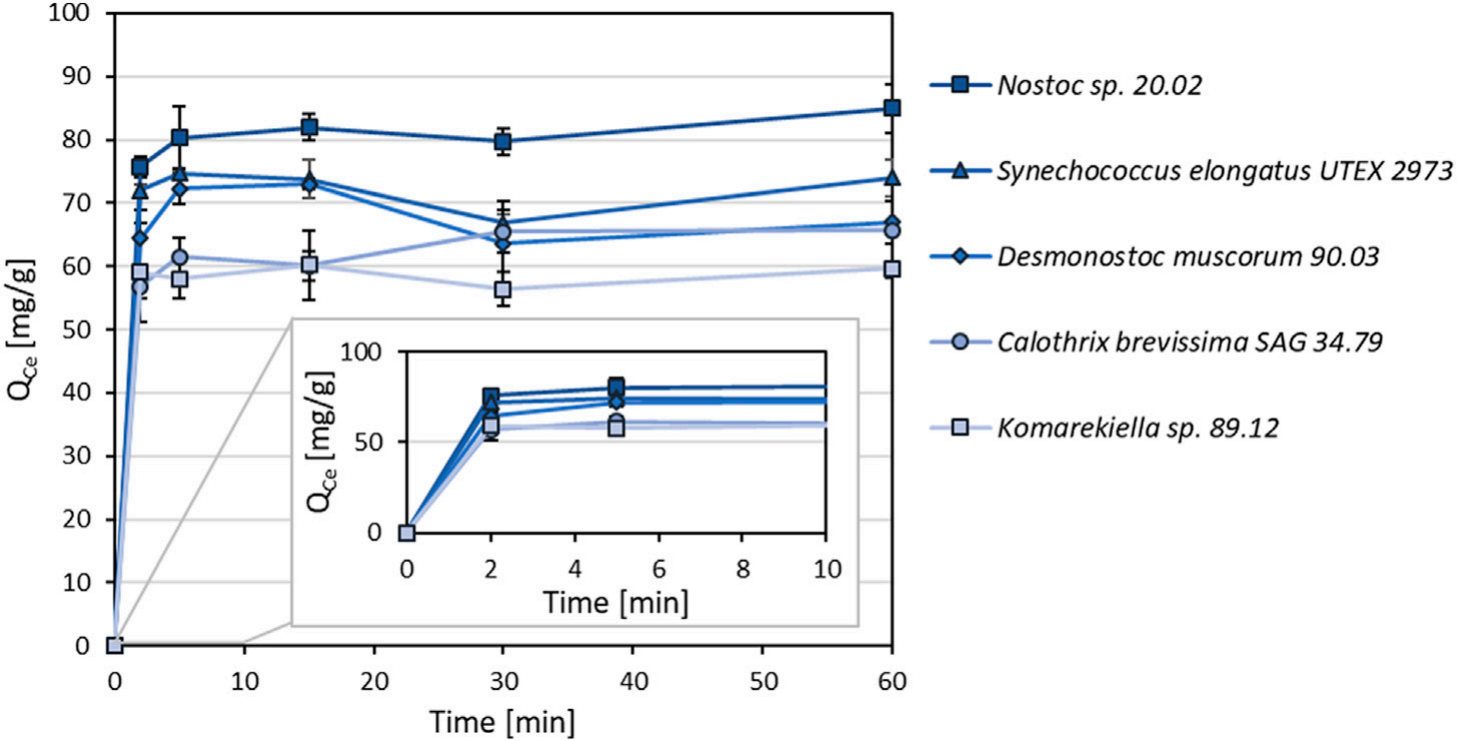
Adsorption kinetics of different cyanobacterial biomasses for Ce^3+^ (Q_ce_, mg Ce^3+^ g^-1^ dry mass) with incubations times between 2–60 min (n = 3).

As shown in Figure 4, metal adsorption for cerium (Ce^3+^) to all tested cyanobacterial biomasses occurred rapidly. The adsorption capacity equilibrium was reached within an incubation time of 5 minutes. After this time, there was no significant change in adsorption capacity within 60 min.

### 3.5 Adsorption isotherms

**FIGURE 5:**
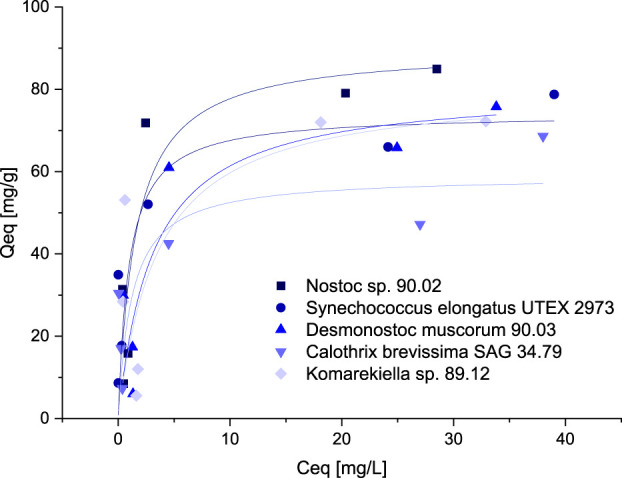
Isotherms for the adsorption of Ce^3+^ (adsorption capacity Q_eq_, mg Ce^3+^ g^-1^ dry mass *versus* Ce^3+^-concentration C_eq_, mg L^-1^) with biomass of five different Cyanobacteria, data points were fitted according to the Langmuir-model.

For the intended application of cyanobacterial biomass for the removal of metals from wastewater, high sorption capacities at relatively low metal concentrations are beneficial. Sorption capacities for the biomass of five selected cyanobacteria species were investigated at concentrations between 0.5–10 mM. The resulting data points were fitted according to the Langmuir and Freundlich model. The best correlation was achieved using the Langmuir model (Figure 5). Although the overall correlation with the model was weak, maximum adsorption capacities predicted by the model are in accordance with the values determined during the screening experiments (Supplementary Tables S1, S2). Adsorption capacities for all tested cyanobacteria exhibited a steep increase at lower equilibrium metal concentrations, showing that sequestration of metals is possible even at low concentrations.

### 3.6 Binding specificity

**FIGURE 6:**
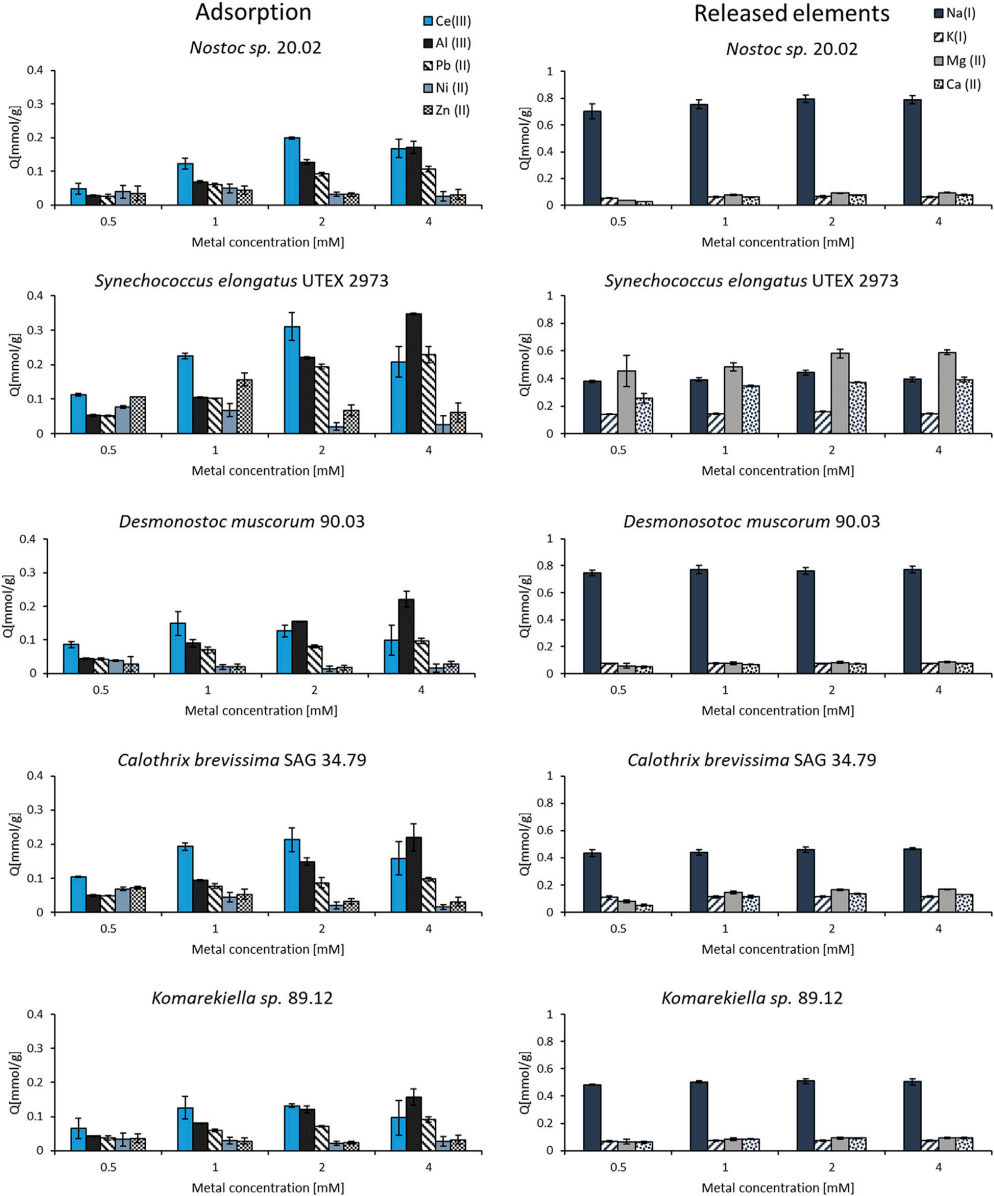
Adsorption and release of elements from biomass incubated in equimolar mixed-metal (Ce, Al, Pb, Ni, and Zn) solutions with concentrations between 0.5–4.0 mM (n = 3).

The binding specificity of cyanobacterial biomass towards cerium was determined in adsorption experiments with other metals (Al, Pb, Ni, and Zn) in equimolar solutions. Starting with a concentration of 0.5 mM, the adsorption capacities were investigated for increasing metal concentrations up to 4 mM. The experiments showed that all elements could be adsorbed by the tested biomass. However, the metal uptake for some elements varied strongly amongst the tested metals (Figure 6). The adsorption capacity for zink and nickel was the lowest, whereas for cerium, the tested biomass showed the highest adsorption capacity in solution with a concentration of 0.5–2.0 mM. Nevertheless, the adsorption capacity for cerium in mixed metal solutions was significantly lower compared to experiments with single-element solutions in previous experiments. This indicates a competition of different elements for the same, limited binding sites on the biomass. The metal uptake of aluminum and lead steadily increased with rising metal concentrations. At a metal concentration of 4 mM, these elements even seemed to replace cerium as the binding capacity for this element dropped for all tested cyanobacteria biomasses. The analysis of metal concentrations via ICP-OES revealed a release of alkaline and alkaline earth metals (Na, K, Mg, and Ca) during the adsorption process. The concentration of these elements increased after incubating the biomass in the equimolar metal solutions containing Ce, Al, Pb, Ni, and Zn. By contrast, mixing the biomass with pure demineralized water did not lead to a notable increase in the concentration of alkaline and alkaline earth metals. This indicates an ion-exchange mechanism in which positively charged metal ions bind to the biomass and replace other ions that exhibit a weaker interaction. For all tested cyanobacteria, Na^+^ ions were the most prevalent ions being released. The biomass of *S. elongatus* was an exception, as Mg^2+^ and Ca^2+^ played a more important role in this case.

### 3.7 FT-IR analysis

**FIGURE 7:**
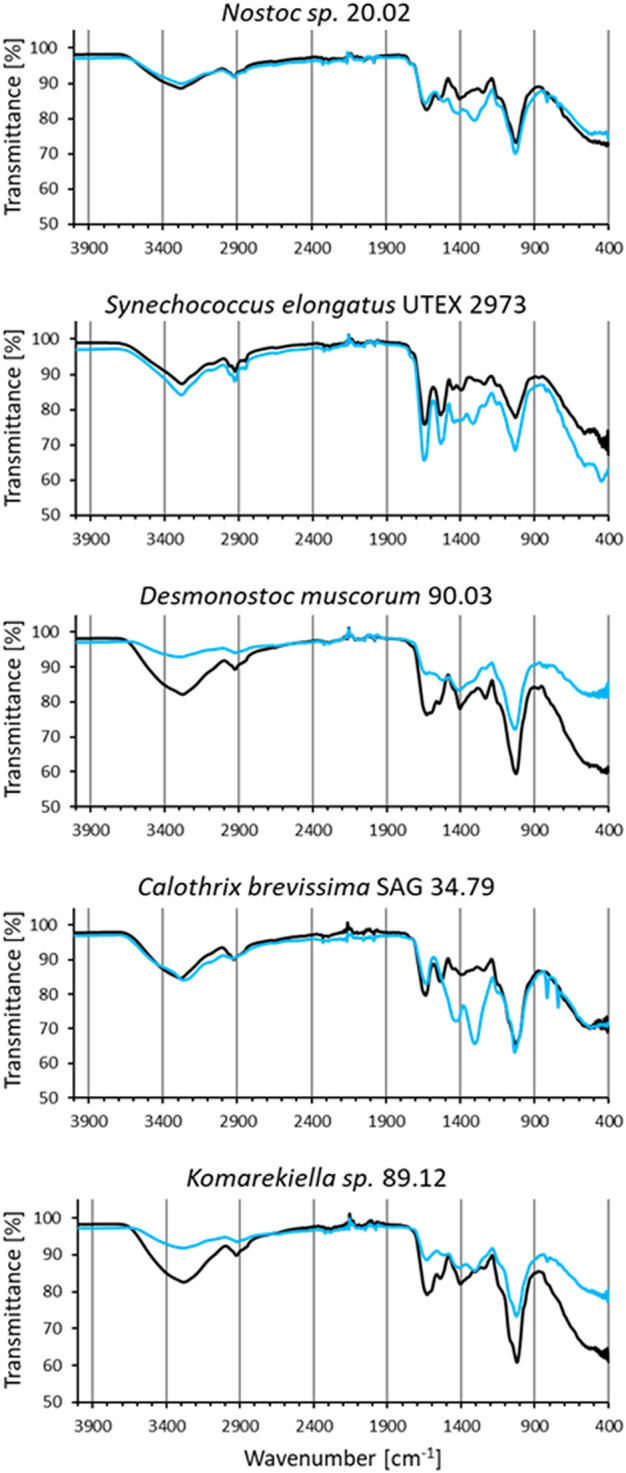
FT-IR spectra of biomass samples before (black) and after incubation in cerium (III) nitrate solution (1 µmol mg^−1^ dry mass) (blue).

IR spectra of all analyzed biomass samples displayed signals that can be assigned to different functional groups (Figure 7). The broad band in the region around 3,350 cm^−1^ in the spectra are linked to the stretching vibrations of hydroxyl groups (Qian et al., 2018), whereas the signal at 2,920 cm^−1^ can be related to the C-H stretching vibrations of CH_2_ groups (Bhattacharya et al., 2014). Signals around 1,630 cm^-1^, which can be assigned to C=O stretching vibrations, indicate the presence of carboxyl groups (Qian et al., 2009). The strong signals around 1,040 cm^-1^ can be assigned to C-O stretching vibration in polysaccharides (Nakamoto 2009). FT-IR spectra of biomasses after interaction with cerium (III) nitrate (Figure 7 blue lines) are characterized by changes in intensity and shifts in position of certain bands due to the interaction with the adsorbed metal ions. The first observed change was the attenuation of intensity in the region between 3,600–3,000 cm^–1^, indicating a decrease of free hydroxyl groups in the biomass (Mitic-Stojanovic et al., 2011). This was most prominent in biomass samples from *D. muscorum* 90.03 and *Komarekiella sp*. 89.12. Likewise, changes in intensities around 1,630 cm^-1^ and 1,040 cm^-1^ indicate an interaction with carboxyl groups (Qian et al., 2009). These changes were more profound for *S. elongates* UTEX 2973, *D. muscorum* 90.03, and *Komarekiella sp*. 89.12. Distinct changes in signal intensities around 1,410 cm^-1^ and 1,290 cm^-1^, which can be observed in samples of *Nostoc sp*. 20.02, *S. elongates* UTEX 2973, and *C. brevissima* SAG 34.79, might be linked to an interaction with aromatic C-C groups and C-O or C-N groups respectivly (Theivandran et al., 2015).

## 4 Discussion

### 4.1 Phylogenetic and taxonomical remarks

In the broad context of biotechnology, cyanobacterial strains are often used without respecting their ecological niche. This is a problem, because some taxa e.g. from aquatic habitats, often cannot be used during biotechnological processes that involve heat or desiccation, while others, such as terrestrial strains, are better candidates and *vice versa*. In addition, it happens quite often that results are not linked to strain identifiers or to wrongly identified taxa what can lead to an incorrect comparison and interpretation of data—a mistake that can remain uncorrected over decades (e.g., Jung et al., 2021b). For these reasons we respected the ecology of the strains used in this study and depicted the phylogenetic placement of the strains. This creates a transparent background for the cyanobacterial strains that we used and allows others to better compare their results. Besides publicly available cyanobacterial strains with a clarified identity, several new isolates were phylogenetically analyzed during this work based on their 16S rRNA gene region (Figure 1). Among these were, for example, the heterocytous, false-branching strain *S. hyalinum* 02.01 that joined the large *S. hyalinum* cluster as outlined by Johansen et al., (Johansen et al., 2017). In addition, the two true-branching, heterocytous strains *Symphyonema bifilamentata* 97.28 and *Reptodigitus sp*. 92.1 were included in the study in order to complement the setup of heterocytous, branching cyanobacteria. The strain 97.28 was treated as *Fisherella ambigua* for the last 50 years of biotechnological research on secondary metabolites but was recently re-assigned as the type strain of the genus *Symphyonema* (Jung et al., 2021b). This strain has great biotechnological potential, because it grows fast and produces a diverse set of secondary metabolites, such as various ambigols (summarized in (Jung et al., 2021b)). The strain 92.1 was formerly treated as *Nostochopsis lobatus,* but doubts about this assignment arose because *N. lobatus* is only known from aquatic habitats. Recently, the new genus *Reptodigitus* was emerged, and the authors pointed out that strain 92.1 needs to be correctly described as a novel *Reptodigitus* species (Casamatta et al., 2020) which the authors of this study will carry out in a follow up study. In contrast to the above named strains, which are low producers of EPS (extracellular polymeric substances), the genus *Komarekiella* and related genera are well known to produce cells and filaments covered by thick EPS sheaths (Scotta Hentschke et al., 2017; Soares et al., 2021). EPS might play a role in metal adsorption (e.g. Al Amin et al., 2021). However, the two strains investigated here are the first strains of this genus described from a desert environment, while the other species of the genus have multiple origins, including lichen symbioses (Jung et al., 2021a; Soares et al., 2021; Panou and Gkelis 2022). All of them have a very complex life cycle in common that can hamper biotechnological applications due to different metabolic activity depending on the developmental stage of the culture. Also, the two strains 90.01 and 89.12 will be described as new species in the future. More challenging to interpret are the phylogenetic and taxonomical positions of *Nostoc* sp. 20.02 and *C. brevissima* SAG 34.79 (Figure 1). The strain 20.02 was isolated as an epiphyte on a cyanolichens and can be considered as a *Nostoc* strain not involved in the symbiosis because most true *Nostoc* lichen photobionts usually join distinct *Nostoc* ‘photobiont clusters’ based on their 16S rRNA (O'Brien et al., 2005). The overall taxonomic position of this strain remains unsure as it also does not cluster within the *Nostoc sensu stricto* clade. Similar uncertainties affect strain SAG 34.79 that could be assigned to *C. brevissima* based on its morphology and phylogenetic position, although there is no cohesive cluster formed and no type strain for the genus deposited. Closely related strains such as *Tolypothrix tenuis* SAG 94.79, *Scytonema mirabile* SAG 83.79, and *T. tenuis* J1 (Figure 1) need further investigation to clarify the state of the genus. *Calothrix* exhibits a notorious morphological heterogeneity and extreme polyphyly, which is evident from various independent clades in the phylogenetic trees of past research [reviewed in (Nowruzi and Shalygin 2021)]. However, even if no phylogenetic or habitat correlation with adsorption capacity could be found, biotechnological studies of cyanobacterial strains should be more often accompanied with phylogenetical studies applying the current standard for taxonomical classification by the so called polyphasic approach (Komárek et al., 2014) to identify taxonomic rearrangements and to avoid confusion regarding species names and strain names from culture collections for biotechnology.

### 4.2 Metal adsorption experiments

For microalgae, the bioremediation, bioaccumulation, or biosorption of common heavy metals such as Pb, Cd, Cr, As, Hg, Ni, etc. is often studied [e.g. (Ahuja et al., 1999; Ç etinkaya Dönmez et al., 1999; Mehta and Gaur 2005)]. The mechanisms behind these adsorption processes vary with species and environmental conditions (Kumar et al., 2015). However, different mechanisms are discussed, such as ion exchange, complexation, electrostatic attraction, and micro-precipitation (Kumar et al., 2015; Yadav et al., 2021). In contrast, the biosorption of REE is studied less. For the adsorption process of REE, the results in this study indicate an ion-exchange mechanism in which cations of alkaline and alkaline earth metals (Na, K, Mg, and Ca) are replaced by other metal cations during the biosorption process with cyanobacterial biomass (Figure 6). This is in agreement with previous experiments using biomass of different microorganisms (Crist et al., 1994; Matheickal et al., 1997; Sulaymon et al., 2013; Liang and Shen 2022). Ion exchange has been proposed as a dominant mechanism during biosorption (Chen et al., 2002; Iqbal et al., 2009). Apart from *Synechocococcus elongates* UTEX 2973 biomass*,* sodium was the predominant element during the ion exchange process. This differs from previous reports in which cations of earth alkaline metals were released in higher percentages (Iqbal et al., 2009; Sulaymon et al., 2013). Additionally, studies reported the replacement of protons with metal cations leading to a decrease in pH during the sorption process (Mashitah et al., 1999; Vasudevan et al., 2002). However, this aspect was not focused on in the experimental setup of this study. The strong influence of pH value on metal uptake shown in this study further emphasizes the correlation between charges on the surface of the biosorbent and the adsorbed metal ions. In previous studies, the effect of pH value on biosorption has been confirmed (García-Rosales et al., 2012; Abdel-Aty et al., 2013). At low pH values, functional groups on the cell surface are either neutral or positively charged. Carboxyl groups for instance are protonated at pH values below 3, whereas amino groups are protonated at pH 4.1 (Eccles 1999). As similar charges create a repulsive force, positive charges on the biomass surface repel metal cations, leading to poor metal uptake at low pH values. Previous studies described a strong influence of hydroxyl and carboxyl groups on the adsorption process for different biomasses (Gupta and Rastogi 2008; Luo et al., 2010; Utomo et al., 2016). Experiments on adsorption kinetics showed a quick metal uptake for all tested biomasses, reaching equilibrium within only a few minutes. In general, the process of metal cations attaching to adsorbents with a mesoporous surface involves two stages (Zinicovscaia et al., 2021). Specifically, the steps involve the migration of ions from the main solution to the boundary layer surrounding the intermediate-pore matrices, and the attachment of the metal ions to the active sites of the adsorbent material *via* adsorption. Previous studies have reported fast kinetics for the adsorption of metals on biomass of other green algae and cyanobacteria (Klimmek et al., 2001). On the other hand, experiments in other studies resulted in incubation times of up to 60 min and more before reaching the maximum adsorption equilibrium (Ahuja et al., 1999; Zinicovscaia et al., 2017). Fast metal uptake is a beneficial factor for the process development beyond laboratory scale as long incubation periods can be avoided, and higher flow rates can be achieved. Adsorption experiments with equimolar mixed-metal solutions were carried out, revealing a preference for certain elements influenced by the total metal concentration. The tested biomasses showed the highest overall adsorption capacity for Ce^3+^ at low metal concentrations. However, cations of these elements were replaced by Pb and Al at higher metal concentrations (2–4 mM) in this experimental setup. Zn and Ni showed to lowest affinity to the tested biomasses. Similar results have been reported for biomass of other microorganisms (Klimmek 2003; Wilke et al., 2006; Huang et al., 2018). At present, our ability to make predictions on binding specificity based on single-element adsorption experiments is limited (Wilke et al., 2006). Regarding a potential industrial application for the recovery of REE, these are promising results, as metal concentrations usually are lower than the highest concentrations in the experimental setup of this study. Furthermore, it should be considered that this study predominately focused on the adsorption of the element cerium. Due to high chemical similarities between REE, it is likely that the adsorption properties of the tested biomasses will be similar for other elements of this group. Nevertheless, additional experiments with other REE are advisable. Target elements could be extracted from the resulting metal-loaded biomass in follow-up processes. The destructive recovery by combustion, resulting in metal-enriched ash, is a simple method with the drawback of losing the initial biomass. An economically more desirable approach is the targeted desorption of elements from loaded biomass, enabling the recycling of the biosorbent. Previous studies have tested various approaches using different acids or complexing agents (Gong et al., 2005; Abdolali et al., 2015). Unfortunately, the adsorption properties of biosorbents are impaired over the curse of a few cycles (Hammaini et al., 2007). Future studies should address the binding specificity and durability of biosorbents to implement biosorption in industrial processes successfully. In competitive systems, the adsorption of different metal cations on biomass is influenced by functional groups on the cell surface. The interaction between metal cations and functional groups still requires more research. According to the current state of knowledge, various ionic properties of metal cations, such as electronegativity, redox potential, and ionic radius can influence the adsorption on biomass (Naja et al., 2010). Depending on the biomass and physico-chemical conditions, multiple mechanisms may be involved in metal sorption simultaneously (Gadd 2009). With respect to different cyanobacterial strains, FT-IR analysis indicated the involvement of various functional groups during like hydroxyl or carboxyl groups during metal adsorption. However, at present, there is no discrete chemical entity that has been identified as dominant cell wall feature that governs metal binding. In a previous study, for instance, it was shown that complex polymeric sugars are involved in the adsorption of terbium by *C. brevissima* (Jurkowski et al., 2022). Cell wall-derived binding entities most likely vary for every organism and metal presented.

## 5 Conclusion

In this study, a diverse group of 12 cyanobacteria was investigated for their potential in the enrichment of REE in a biosorption process. Metal uptake varied strongly among the tested strains, with *Nostoc* sp. 20.02 showing the highest maximum adsorption capacity of 84.2–91.5 mg g^-1^. However, there was no apparent correlation between maximum adsorption capacity and phylogenetic relationship nor for the ecological habitat of the strains. This could be explained by variations in the composition of metal interacting functional groups located at the cell surface. Moreover, many cyanobacteria that showed high adsorption capacities for REE produce extracellular polymeric substances (EPS) that are known to facilitate metal adsorption (Pagliaccia et al., 2022). The composition of these EPS and their influence on the adsorption of REE should be further investigated in future studies. The determination of relevant parameters for improving the metal uptake revealed a pH optimum at 5 to 6 and fast adsorption kinetics reaching adsorption equilibrium within an incubation time of a few minutes. In addition, metal analysis strongly indicated an ion-exchange mechanism during the biosorption process in which Na^+^, K^+^, Mg^2+^, and Ca^2+^ ions are replaced by metal cations that bind to the surface of the biomass. These observations are in accordance with previous studies that were conducted on algal, bacterial, and other biomasses (Acheampong et al., 2011; Sulaymon et al., 2013; Liang and Shen 2022). The isolation of single target elements in a technical biosorption process remains a challenging task due to the complex surface structure and the heterogeneity of functional groups. Nevertheless, based on the results of this study, the enrichment of metal elements from diluted solutions is possible. For the development of an industrial process, parameters need to be further optimized and adjusted depending on the metal composition in the wastewater and the biomass that is used as biosorbent.

## Data Availability

The original contributions presented in the study are included in the article/Supplementary Material, further inquiries can be directed to the corresponding author. The 16S rRNA gene sequences generated during this study were added to NCBI GenBank stated by their accession number in the phylogenetic tree (Figure 1).
