# Supplementary material for: Rare earths stick to rare cyanobacteria: Future potential for bioremediation and recovery of rare earth elements
Source: Front Bioeng Biotechnol. 2023 Feb 28;11:1130939. doi: 10.3389/fbioe.2023.1130939 (PMC10011134; doi:10.3389/fbioe.2023.1130939)
Supplement: Supplementary file 1 [file Table1.DOCX]

Supplementary Material

**Rare Earths stick to rare Cyanobacteria: Future Potential for Bioremediation and Recovery of Rare Earth Elements**

**Michael Paper^1 †^, Max Koch^2 †^, Patrick Jung^3 †^, Michael Lakatos^3^, Tom Nilges^2^, Thomas B. Brück^1,4*^**

^1^ Werner Siemens-Chair of Synthetic Biotechnology, School of Natural Sciences, Dept. of Chemistry, Technical University of Munich, Garching, Germany

^2^ Synthesis and Characterization of Innovative Materials, School of Natural Sciences, Dept. of Chemistry, Technical University of Munich, Garching, Germany

^3^ Integrative Biotechnology, University of Applied Sciences Kaiserslautern, Pirmasens, Germany

^4^ TUM AlgaeTec Center, Ludwig Bölkow Campus, Department of Aerospace and Geodesy, Taufkirchen, Germany

***Correspondence:
Thomas B. Brück** − Werner Siemens-Chair of Synthetic Biotechnology, School of Natural Sciences, Dept. of Chemistry, Technical University of Munich, D- 85748 Garching, Germany; orcid.org/0000-0002-2113-6957; Phone: +49 89-28913253; Email: [brueck@tum.de](mailto:brueck@tum.de)

† These authors contributed equally to this work.

Supplementary Table S1: Maximum adsorption capacity (Q_max_, mg Ce^3+^ g^-1^ dry mass) and Langmuir adsorption coefficient (K) of the Langmuir model for the adsorption of Ce^3+^ by different cyanobacteria (n=3)

|  | Qmax | K | R^2^ |
| --- | --- | --- | --- |
| *Nostoc sp.* 20.02 | 90.0 ± 11.6 | 0.63 | 0.88 |
| *Synechococus elongatus* UTEX 2973 | 74.1 ± 12.4 | 0.76 | 0.71 |
| *Desmonostoc muscorum* 90.03 | 80.6 ± 13.5 | 0.32 | 0.83 |
| *Calothrix brevissima* SAG 34.79 | 58.9 ± 11.6 | 0.82 | 0.68 |
| *Komarekiella sp.* 89.12 | 80.8 ± 25.2 | 0.28 | 0.53 |

Supplementary Table S2: Adsorption capacity constant (K_f_) and intensity constant (n) of the Freundlich model for the adsorption of Ce^3+^ by different cyanobacteria (n=3)

|  | K_f_ | n | R^2^ |
| --- | --- | --- | --- |
| *Nostoc sp.* 20.02 | 33.0± 9.8 | 3.4 | 0.74 |
| *Synechococus elongatus* UTEX 2973 | 38.3± 6.3 | 5.3 | 0.84 |
| *Desmonostoc muscorum* 90.03 | 24.2± 8.5 | 3.1 | 0.74 |
| *Calothrix brevissima* SAG 34.79 | 27.2± 6.9 | 2.2 | 0.72 |
| *Komarekiella sp.* 89.12 | 25.7± 11.4 | 3.3 | 0.52 |
